# Association of gamma-tocopherol serum concentrations and blood pressure among adults in the United States: a cross-sectional study

**DOI:** 10.3389/fcvm.2023.1182731

**Published:** 2023-06-19

**Authors:** Zhijian Wu, Feng Xie, Kai Li, Jie Feng, Leilei Han, Yanqing Wu

**Affiliations:** Department of Cardiology, The Second Affiliated Hospital of Nanchang University, Nanchang, China

**Keywords:** blood pressure, hypertension, gamma-tocopherol, vitamin E, alcohol consumption

## Abstract

**Background:**

hypertension is one of the major preventable risk factors for numerous diseases. The role of vitamin E in blood pressure (BP) has been controversial. We aimed to investigate the relationship between gamma-tocopherol serum concentration (GTSC) and BP

**Methods:**

Data from 15,687 US adults from the National Health and Nutrition Examination Survey (NHANES) were analyzed. The correlations of GTSC with systolic BP (SBP), diastolic BP (DBP), and prevalence of hypertension were investigated by multivariate logistic regression models, generalized summation models, and fitted smoothing curves. Subgroup analyses were performed to investigate possible effect modifiers between them.

**Results:**

With each natural log increase in GTSC, SBP, and DBP increased by 1.28 mmHg (*β* 1.28, 95% CI 0.71–1.84) and 1.15 mmHg (*β* 1.15, 95% CI 0.72–1.57), respectively, both *P* for trend < 0.001; the prevalence of hypertension increased by 12% (OR 1.12, 95% CI 1.03–1.22), *P* for trend 0.008. In subgroup analysis, in drinkers, with each natural log increase in GTSC, SBP, and DBP increased by 1.77 mmHg (*β* 1.77,95% CI 1.13–2.41) and 1.37 mmHg (*β* 1.37,95% CI 0.9–1.85), respectively, whereas they were not correlated in non-drinkers.

**Conclusion:**

GTSC was linearly and positively associated with SBP, DBP, and the prevalence of hypertension, and alcohol consumption may modify the relationship of GTSC with SBP and DBP.

## 1. Introduction

In recent decades, hypertension has been the major single factor in all causes of death and disability worldwide and is one of the major preventable risk factors for numerous diseases such as cardiovascular disease (CVD), renal insufficiency, and Alzheimer’s disease (1). In the United States, hypertension is the largest contributor to CVD deaths compared to other modifiable risk factors and is second only to smoking in terms of all-cause mortality (2). The progression of disease due to blood pressure (BP) is known to be graded and continuous, and a BP of 115/75 mmHg or less is considered to be within the perfectly normal range (3). The Global Burden of Disease Study shows that 9.4 million deaths and 212 million lost healthy life yearly due to exceeding optimal BP levels. It is worrying that over 3.5 billion people worldwide have systolic BP (SBP) outside the ideal range (i.e., >115/mmHg) and 874 million people have SBP pressure above 140 mmHg, and this data will gradually increase with economic and social development (4). Therefore, controlling BP is an important step in reducing the burden of disease and increasing the life expectancy of the world’s population.

Many factors have been shown to be associated with BP increase, such as socio-demographic factors like gender, age, ethnicity, and environmental and behavioral factors. There are also many changeable exposure elements such as a high sodium diet, low potassium diet, obesity, and a sedentary lifestyle that could increase BP (5). In addition, studies have shown that oxidative stress plays an important role in the development of hypertension (6–8). Oxidative stress increases the generation of endothelium-derived contractile factors and decreases the biological availability of nitric oxide, resulting in impairment of vascular relaxation and endothelial dysfunction in hypertensive patients (9). Vitamin E is an antioxidant, however, its effect on BP has been controversial. Some interventional trials and animal experiments indicated that vitamin E could produce beneficial effects on BP by inhibiting oxidative stress (10–12). Some studies also argue that vitamin E has no effect on BP and may even be harmful (13–16). In addition, it has recently been shown that the effect of vitamin E on BP has a multisegmented effect, with an inverted J-shaped relationship (17). Differences in study design, sample size, ethnic distribution, and control of confounding factors may explain the controversial results among these studies. More importantly, most of these studies did not separately investigate the single active form of vitamin E.

Vitamin E is made up of tocopherols and tocotrienols (*α*, *β*, *γ*, and *δ*) and has two main dietary forms in the body, α-tocopherol (ATC) and γ-tocopherol (GTC), of which the most studied is ATC, because ATC is most bioactive and abundant in the blood, and vitamin E deficiency can be corrected by taking this supplement (18). More recently, there has been a growing body of research discussing the role of GTC in public health. GTC is the most common form of vitamin E in the diet, and its concentration in tissues is much greater than its concentration in the blood (19, 20). Differences in morphology, biological activity, and tissue distribution lead to different biological effects of ATC and GTC. Because vitamin E may have an impact on the development of hypertension and there are few studies on the role of GTC, we propose to conduct cross-sectional studies in U.S. adults to explore the correlation between GTC serum concentration (GTSC) and BP, including SBP, diastolic BP (DBP), and the prevalence of hypertension.

In this study, we extracted data from a representative National Health and Nutrition Examination Survey (NHANES) database of 4 cycles with GTSC data to investigate the correlation between GTSC and BP, and possible modifiers of this relationship.

## 2. Method

### 2.1. Study design and population

The data used in this study were all from four cycles in the NHANES database. The NHANES is a continuous representative survey of the U.S. national population that provides a wealth of data on the nutrition and health of adults and children in the U.S. using a complex, multi-stage, probability sampling design. The study was approved by the Ethics Review Committee of the National Center for Health Statistics and with the written consent of each participant. More information can be found at https://www.cdc.gov/nchs/nhanes/index.htm.

**Figure 1:**
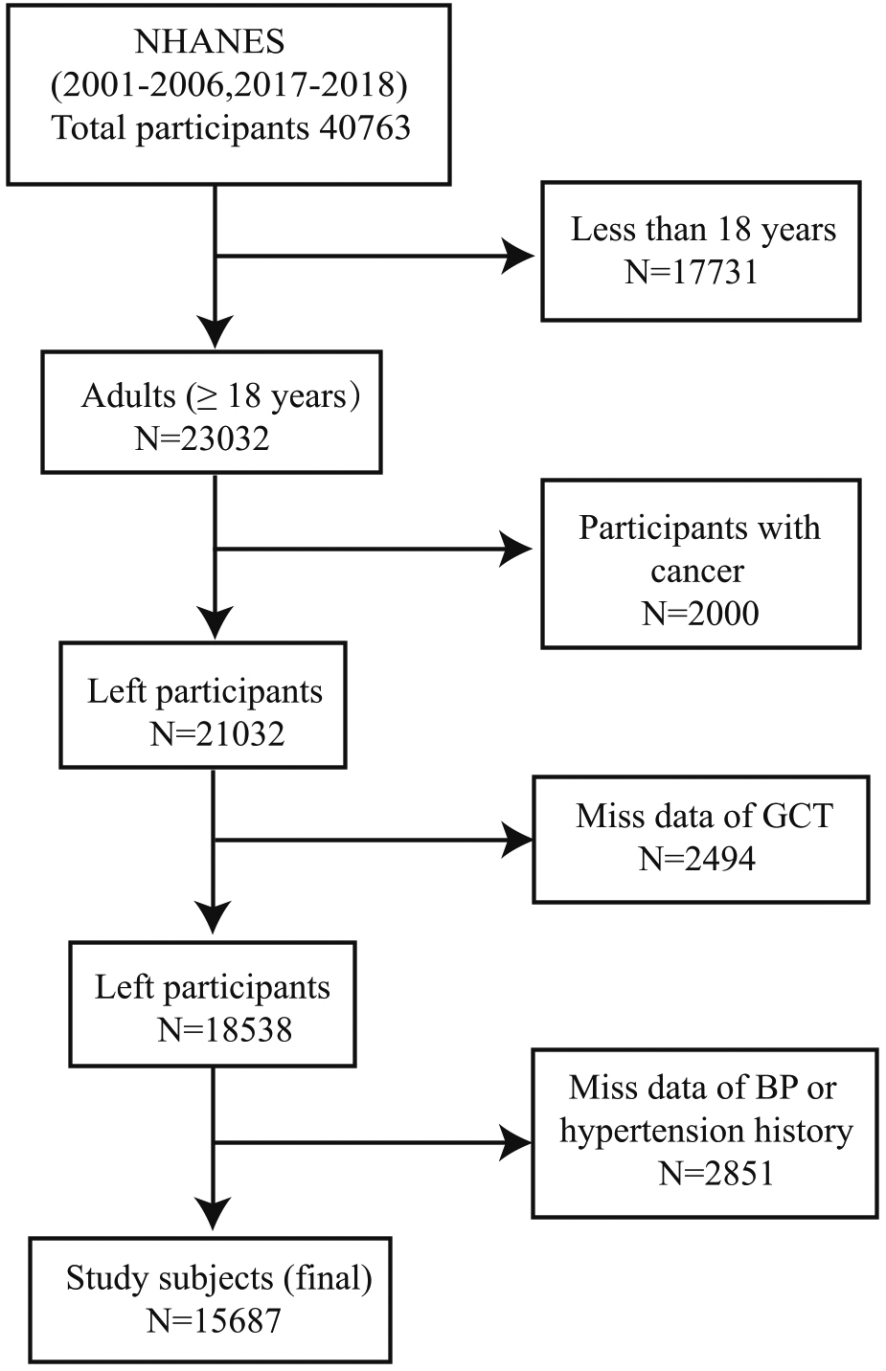
Flow chart of participants.

We performed this cross-sectional study using information from participants aged ≥18 years (*n* = 23032) in the NHANES (2001–2002, 2003–2004, 2005–2006, 2017–2018) study. Exclusion criteria are as follows: participants with cancer (*n* = 2000), patients with missing GTSC data (*n* = 2494), and participants with missing BP data or hypertension history (*n* = 2851). Finally, 15,687 participants were included in the statistical analysis (Figure 1).

### 2.2. The exposure and outcome variables

No fasting or special diet is required before the blood was collected by a phlebotomist. The exposure variable was the GTSC, which was measured by high-performance liquid chromatography with multiwavelength photodiode-array absorbance detection. Tocopherols have absorption maxima between 292 and 300 nm and chromatograms are recorded using a computer data system. Spectrophotometric methods are used for quantitative analysis. GTSC was a skewed distribution in our study, and we convert it to a natural logarithm with base e (Ln GTSC) in the data analysis. The outcome variables were SBP, DBP, and the prevalence of hypertension. The SBP and DBP were defined respectively as the average of the SBP and DBP measured at three different times. Hypertension was defined as a self-reported diagnosis of hypertension, or SBP ≥ 140 mmHg and/or DBP ≥ 90 mmHg, or the use of antihypertensive medication.

### 2.3. Potential covariates

In our study, covariates included demographic data [sex, age, race, education levels, and poverty income ratio (PIR)], examination data (weight (kg), height (cm), body mass index (BMI, kg/m^2^), and waist (cm)), laboratory data (Albumin (g/L), blood urea nitrogen (BUN, mmol/L), uric acid (UA, umol/L), serum creatinine (Scr, mol/L), estimated glomerular filtration rate (eGFR, we calculated by the modification of diet in renal disease equation, ml/min/1.73 m^2^), fasting blood glucose (FBG, mmol/L), glycated haemoglobin A1c (HbA1c, %), total cholesterol (TC, mmol/L), triglycerides (TG, mmol/L), high-density lipoprotein cholesterol (HDL-C, mmol/L)), and questionnaire data (History of diabetes, heart failure (HF), coronary artery disease (CAD), smoking, and drinking). Diabetes was defined as a self-reported diagnosis of diabetes or having an HbA1c level ≥ 6.5%, or FPG ≥ 7 mmol/L. HF and CAD were defined as the self-reported diagnosis of HF and CAD, respectively.

### 2.4. Statistical analysis

Described continuous data by mean ± standard deviations (SDs) or median (interquartile ranges, Q1–Q3) and categorical data by number (%), respectively. Differences between the tertiles of Ln GTSC groups were compared using one-way ANOVA for continuous variables and the chi-square test for categorical variables. In multivariate logistic regression models, covariates were proven traditional or suspected risk factors for hypertension, or the Ln GTSC estimates for SBP, DBP, and hypertension changed by more than 10% (21). We investigated the relationship between GTSC (continuous and categorical) and BP values and the prevalence of hypertension using multivariate logistic regression analysis. Model 1 represents unadjusted data. Model 2 adjusts for demographic information: age, sex, race, education level, and PIR. Model 3 additionally adjusts for smoking status, drinking status, diabetes, CAD, HF, BMI, albumin, BUN, UA, Scr, eGFR, FBG, HbA1c, TC, TG, and HDL-C. The shape of the relationship between GTSC and BP and the prevalence of hypertension, respectively, was described by using the generalized additive model (GAM) and smoothed curve fit (penalized spline method). Subgroup analysis using stratified multiple regression analysis for the following variables: age (<60 vs. ≥60 years), sex (male vs. female), race (Mexican American vs. Other Hispanic vs. Non-Hispanic White vs. Non-Hispanic Black vs. other races), BMI ((<24 vs. ≥ 24 kg/m^2^), CAD (yes vs. no), HF (yes vs. no), diabetes (yes vs. no), smoking status (yes vs. no), drinking status (yes vs. no), and eGFR (<60 vs. ≥60 ml/min/1.73 m^2^).

All analyses were conducted with package R (http://www.R-project.org) and EmpowerStats (http://www.empowerstats.com), with a *P* value <0.05 considered statistically significant.

## 3. Results

### 3.1. Baseline participant characteristics

Table 1 presents the baseline characteristics of all participants and participants grouped by the tertiles of GTSC. Overall, 15,687 participants with a mean age of 44 ± 18.97 years were included in our study, and 7,629 (48.63%) were males. Among all participants, 5,711 (36.41%) had hypertension and the mean Lg GTSC was 1.50 ± 0.57 umol/L. In the three GTSC groups, differences were statistically significant for all variables except sex. The participants in the GTSC T3 group were likely to be younger, non-Hispanic black; have hypertension, diabetes, HF, CAD; be smokers and non-drinkers; have the higher level for SBP, DBP, weight, BMI, waist circumference, BUN, UA, Scr, FBG, HbA1c, TC and TG; and have lower levels of poverty income ratio, eGFR, height, and HDL-C, than those in the lower GTSC group.

**Table 1:** Baseline characteristics of study participants.

Characteristics^a^	GTSC^b^ (umol/L) tertiles				
	Total	Tertiles 1	Tertiles 2	Tertiles 3	*P*-value
GTSC^b^ rang	−1.78–4.32	−1.78–1.30	1.31–1.76	1.76–4.32	
N	15687	5198	5245	5244	
Sex					0.341
female	8058 (51.37%)	2689 (51.73%)	2651 (50.54%)	2718 (51.83%)	
male	7629 (48.63%)	2509 (48.27%)	2594 (49.46%)	2526 (48.17%)	
Age (years)	45.21 ± 18.97	47.54 ± 20.04	42.21 ± 18.58	45.87 ± 17.85	<0.001
Race					<0.001
Mexican American	3179 (20.27%)	1024 (19.70%)	1078 (20.55%)	1077 (20.54%)	
Other Hispanic	823 (5.25%)	313 (6.02%)	319 (6.08%)	191 (3.64%)	
Non-Hispanic White	6940 (44.24%)	2444 (47.02%)	2158 (41.14%)	2338 (44.58%)	
Non-Hispanic Black	3402 (21.69%)	824 (15.85%)	1228 (23.41%)	1350 (25.74%)	
Other Race	1343 (8.56%)	593 (11.41%)	462 (8.81%)	288 (5.49%)	
Education level					<0.001
<9th grade	1672 (10.66%)	553 (11.58%)	553 (11.93%)	566 (11.63%)	
9−11th grade	1975 (12.59%)	533 (11.16%)	596 (12.86%)	846 (17.39%)	
High school	3434 (21.89%)	993 (20.80%)	1145 (24.70%)	1296 (26.63%)	
AA degree	4162 (26.53%)	1343 (28.13%)	1392 (30.03%)	1427 (29.33%)	
College or above	3032 (19.33%)	1352 (28.32%)	949 (20.47%)	731 (15.02%)	
PIR	2.60 ± 1.62	2.76 ± 1.65	2.56 ± 1.63	2.47 ± 1.58	<0.001
Hypertension					<0.001
No	9976 (63.59%)	3384 (65.10%)	3566 (67.99%)	3026 (57.70%)	
yes	5711 (36.41%)	1814 (34.90%)	1679 (32.01%)	2218 (42.30%)	
Diabetes					<0.001
no	13592 (86.64%)	4597 (88.44%)	4646 (88.58%)	4349 (82.93%)	
yes	2095 (13.36%)	601 (11.56%)	599 (11.42%)	895 (17.07%)	
Heart failure^c^					0.036
No	13904 (88.63%)	4652 (97.42%)	4533 (97.82%)	4719 (96.98%)	
yes	371 (2.37%)	123 (2.58%)	101 (2.18%)	147 (3.02%)	
Coronary artery disease^c^					<0.001
No	13308 (84.83%)	4425 (92.67%)	4384 (94.61%)	4499 (92.46%)	
yes	967 (6.16%)	350 (7.33%)	250 (5.39%)	367 (7.54%)	
Smoking status^c^					<0.001
none	7872 (50.18%)	2826 (57.77%)	2582 (54.85%)	2464 (50.38%)	
current	6618 (42.19%)	2066 (42.23%)	2125 (45.15%)	2427 (49.62%)	
Drinking status^c^					<0.001
none	3312 (21.11%)	1004 (21.69%)	1054 (23.73%)	1254 (27.04%)	
current	10396 (66.27%)	3625 (78.31%)	3388 (76.27%)	3383 (72.96%)	
SBP (mmHg)	123.43 ± 19.28	122.52 ± 19.55	121.83 ± 18.36	125.93 ± 19.67	<0.001
DBP (mmHg)	70.37 ± 12.88	69.13 ± 12.39	69.88 ± 12.74	72.08 ± 13.30	<0.001
Weight (kg)	80.26 ± 20.42	74.84 ± 18.01	79.84 ± 19.93	86.07 ± 21.60	<0.001
Height (cm)	167.58 ± 10.11	167.03 ± 10.20	167.94 ± 10.12	167.79 ± 10.01	<0.001
BMI (kg/m2)	28.51 ± 6.52	26.75 ± 5.57	28.25 ± 6.40	30.53 ± 6.95	<0.001
Waist(cm)	97.20 ± 16.04	93.05 ± 14.91	96.29 ± 15.70	102.25 ± 16.11	<0.001
Albumin (g/L)	41.67 ± 3.81	41.94 ± 3.75	41.74 ± 3.87	41.36 ± 3.79	<0.001
BUN (mmol/L)	4.71 ± 2.05	4.93 ± 2.11	4.55 ± 1.92	4.65 ± 2.10	<0.001
UA (umol/L)	317.93 ± 85.97	306.53 ± 82.88	315.98 ± 84.14	331.14 ± 88.97	<0.001
Scr (mol/L)	79.28 ± 37.90	78.87 ± 35.22	78.60 ± 41.29	80.36 ± 36.90	0.039
eGFR (ml/min/1.73 m^2^)	95.52 ± 28.51	95.06 ± 29.01	97.59 ± 27.65	93.90 ± 28.75	<0.001
FBG (mmol/L)	5.43 ± 1.86	5.28 ± 1.45	5.32 ± 1.66	5.70 ± 2.33	<0.001
HBA1C (%)	5.60 ± 1.00	5.51 ± 0.79	5.51 ± 0.87	5.76 ± 1.25	<0.001
TC (mmol/L)	5.05 ± 1.11	4.75 ± 1.04	4.92 ± 1.01	5.46 ± 1.15	<0.001
Triglycerides (mmol/L)	1.61 ± 1.47	1.30 ± 0.74	1.43 ± 0.92	2.10 ± 2.16	<0.001
HDL-C (mmol/L)	1.38 ± 0.41	1.43 ± 0.40	1.39 ± 0.40	1.33 ± 0.42	<0.001

GTSC, gamma-tocopherol serum concentration; PIR, poverty-income ratio; SBP, systolic blood pressure; DBP, diastolic blood pressure; BMI, body mass index; BUN, blood urea nitrogen; UA, uric acid; SCR, Serum creatinine; eGFR, estimated glomerular filtration rate; FPG, fasting plasma glucose; HbA1c, hemoglobin A1c, TC total cholesterol; HDL-C, high-density lipoprotein cholesterol.

^a^ Data are presented as mean ± standard deviation or median (Q1–Q3) and numbers (%) as appropriate.

^b^ GTSC value was log e-transformed (e = 2.718).

^c^ Numbers that do not add up to 100% are attributable to missing data.

### 3.2. Association of GTSC with BP and prevalence of hypertension

**Figure 2:**
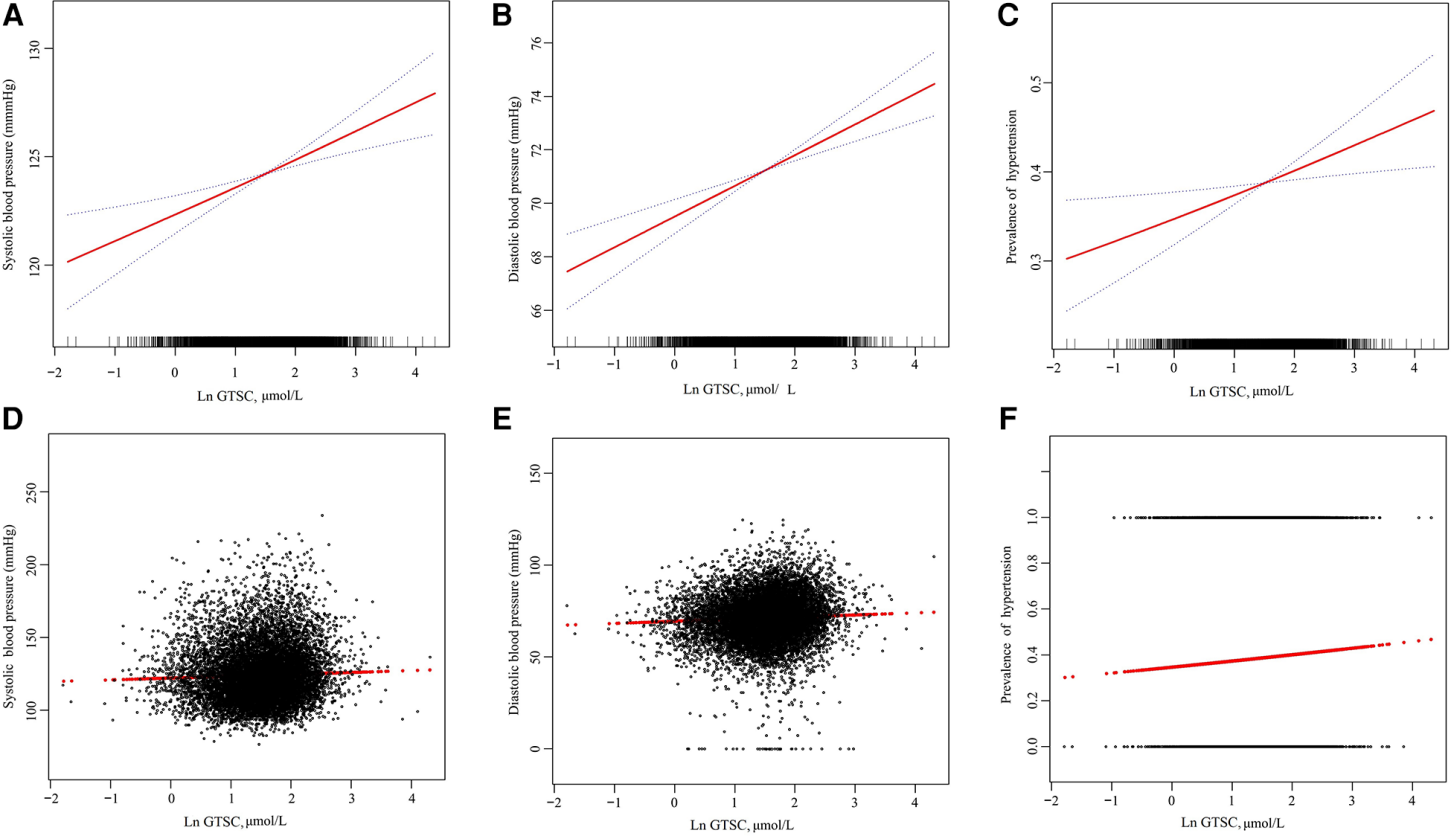
The association of GTSC with SBP (**A,D**), DBP (**B,E**), and prevalence of hypertension (**C,F**). (**A**–**C**): The solid line and dashed line represent the estimated values and their corresponding 95% confidence interval, respectively. (**D**–**F**): Each black point represents a sample. Age, sex, race, education level, and poverty income ratio; smoking status, drinking status, diabetes, CAD, HF, BMI, albumin, BUN, UA, Scr, eGFR, FBG, HbA1c, TC, TG, and HDL-C were adjusted.

As shown in Table 2, in logistic regression, GTSC was significantly and positively associated with BP increase and the prevalence of hypertension in all participants, regardless of whether they were adjusted for confounders. After adjusting for age, sex, race, education level, PIR, smoking status, drinking status, diabetes, CAD, HF, BMI, albumin, BUN, UA, Scr, eGFR, FBG, HbA1c, TC, TG, and HDL-C, each natural log increase in GTSC was associated with a 1.28 mmHg increase in SBP (*β* 1.28, 95% CI 0.71–1.84) and a 1.15 mmHg increase in DBP (*β* 1.15, 95% CI 0.72–1.57), and a 12% increase in the prevalence of hypertension (OR 1.12, 95% CI 1.03–1.22). We grouped GTSC in tertile groups and used the T1 group as the reference group to further evaluate the relationship between GTSC and BP values and the prevalence of hypertension. Compared with the T1 reference group, SBP increased by 0.71 mmHg (*β* 0.71,95%CI −0.01–1.43) and 1.39 mmHg (*β* 1.39,95%CI 0.62–2.15) in the T2 and T3 groups, respectively, *P* for trend <0.001; DBP increased by 0.53 mmHg (*β* 0.53, 95%CI −0.02–1.07) and 1.39 mmHg (*β* 1.39,95%CI 0.82–1.97), respectively, *P* for trend <0.001; and the relative risk of hypertension incidence increased by 8% (OR 1.08,95%CI 0.96–1.21) and 17% (OR 1.17,95%CI 1.04–1.32), *P* for trend 0.008. In summary, SBP, DBP, and the prevalence of hypertension were all linearly and positively correlated with GTSC. The above findings are consistent with the results of the smoothed curve fitting (Figure 2).

**Table 2:** Association of GTSC with SBP, DBP, and the prevalence of hypertension among American adults.

Exposure	SBP (mmHg, β, 95%CI)			DBP (mmHg, β, 95% CI)			Hypertension (OR, 95% CI)		
	Model 1	Model 2	Model 3	Model 1	Model 2	Model 3	Model 1	Model 2	Model 3
Ln GTSC	1.95 (1.42, 2.48)	2.85 (2.36,3.35)	1.28 (0.71, 1.84)	2.29 (1.93, 2.64)	2.50 (2.12, 2.87)	1.15 (0.72, 1.57)	1.19 (1.12, 1.26)	1.45 (1.35, 1.56)	1.12 (1.03, 1.22)
Ln GTSC									
T1	reference	Reference	reference	reference	reference	reference	reference	reference	reference
T2	−0.69 (−1.43, 0.05)	1.60 (0.90, 2.30)	0.71 (−0.01, 1.43)	0.74 (0.25, 1.24)	1.22 (0.68, 1.75)	0.53 (−0.02, 1.07)	0.88 (0.81, 0.95)	1.22 (1.10, 1.35)	1.08 (0.96, 1.21)
T3	3.40 (2.67, 4.14)	3.66 (2.97, 4.36)	1.39 (0.62, 2.15)	2.95 (2.46, 3.44)	3.18 (2.65, 3.71)	1.39 (0.82, 1.97)	1.37 (1.26, 1.48)	1.64 (1.48, 1.81)	1.17 (1.04, 1.32)
*P* for trend	<0.001	<0.001	<0.001	<0.001	<0.001	<0.001	<0.001	<0.001	0.008

GTSC, gamma-tocopherol serum concentration; Ln GTSC was GTSC log e-transformed (e = 2.718); SBP, systolic blood pressure; DBP, diastolic blood pressure. Model 1 adjusts for none; Model 2 adjusts for age, sex, race, education level, and poverty income ratio; Model 3 adjusts for age, sex, race, education level, and poverty income ratio; smoking status, drinking status, diabetes, CAD, HF, BMI, albumin, BUN, UA, Scr, eGFR, FBG, HbA1c, TC, TG, and HDL-C.

### 3.3. Subgroup analyses

**Figure 3:**
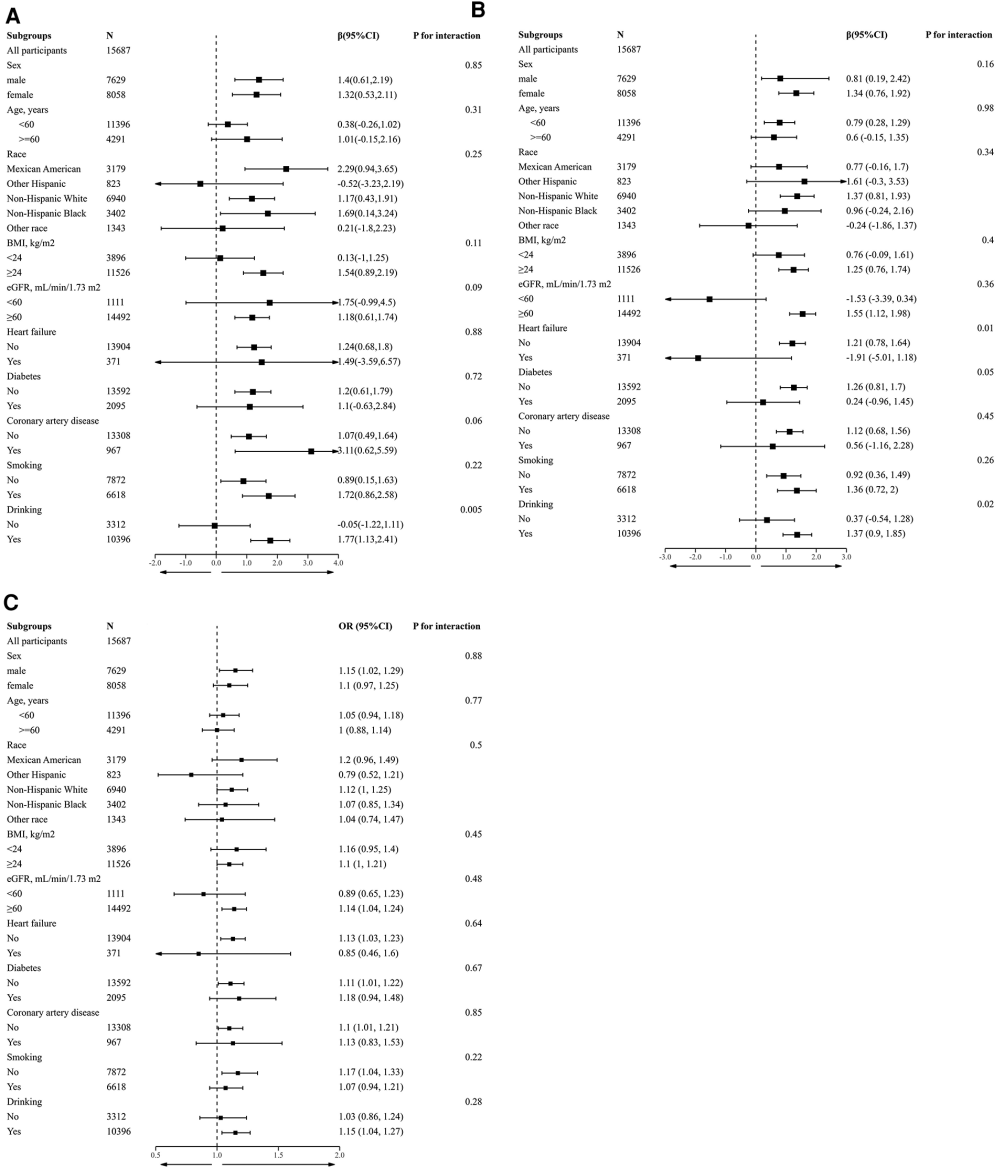
Stratified analyses by potential modifiers of the association between GTSC and SBP (**A**), DBP (**B**), and prevalence of hypertension (**C**). *Each subgroup analysis adjusted for Age, sex, race, education level, and poverty income ratio; smoking status, drinking status, diabetes, CAD, HF, BMI, albumin, BUN, UA, Scr, eGFR, FBG, HbA1c, TC, TG, and HDL-C were adjusted. except for the stratifying variable. *Numbers that do not add up to 100% are attributable to missing data

To further verify the reliability of the results in the presence of confounding factors and whether there are factors that may modify the relationship between GTSC and BP and the incidence of hypertension, we conducted subgroup analyses by stratifying the main covariates. Except for drinking status, there were no other covariates that significantly modified the relationship between GTSC and BP values and the incidence of hypertension, including sex, age, race, BMI, eGFR, HF, CAD, DM, and smoking status (all P-interaction > 0.05) (Figure 3). In individuals who consumed alcohol, each natural log increase in GTSC was associated with an increase in SBP of 1.77 mmHg (*β* 1.77,95% CI 1.13–2.41) and an increase in diastolic BP of 1.37 mmHg (*β* 1.37,95% CI 0.9–1.85). There was no statistically significant relationship between GTSC and BP (either diastolic or systolic) in individuals who did not consume alcohol. There may be an interaction between GTSC and alcohol consumption in terms of BP (SBP: *P* for interaction = 0.005, DBP: *P* for interaction = 0.02), but not in terms of hypertension incidence (*P* for interaction = 0.28) (Figure 3).

## 4. Discussion

In this large sample cross-sectional study, we found that GTSC was positively associated with SBP, DBP, and the prevalence of hypertension, and this relationship remained significant after adjustment for confounders. Furthermore, we noted that alcohol consumption modifies the relationship of GTSC with SBP and DBP, but not the relationship with the incidence of hypertension.

To date, there is still controversy regarding the role of vitamin E in hypertension. Boshtam et al. conducted a randomized triple-blind placebo-controlled trial including 78 patients with mild hypertension in Isfahan and indicated that compared to the placebo group, the oral vitamin E group was able to significantly reduce SBP (−24% in the vitamin E group versus −1.6% in the placebo group) and DBP (−12.5% in the vitamin E versus −6.2% in the placebo group) (*p* < 0.05) (11). Although this study followed a strict clinical randomized study design, it had significant limitations, such as its small sample size, which added significant bias, and weight (kg), which is known to be an important risk factor for hypertension, was different between the two groups (88.91 ± 20.03 vs 75.92 ± 10.67). In addition, there are some cross-sectional studies, meta-analyses, and animal studies that also suggest that taking vitamin E can lower BP (15, 22, 23). However, other studies hold a different view. Ward and his colleagues also performed a randomized, double-blind, placebo-controlled trial including 58 patients with type 2 diabetes to investigate the effects of vitamin E on hypertension (24). Compared with the placebo group, the *α*-tocopherol group (500 mg/day for 6 weeks) increased SBP by 7.0 mmHg (*β* 7, 95% CI 5.2–8.8) and DBP by 5.3 mmHg (*β* 5.3, 95% CI 4.0–6.5); the mixed tocopherol group (60% *γ*-, 25% *δ*- and 15% *α*-tocopherol, 500 mg /day for 6 weeks) increased SBP by 6.8 mmHg (*β* 6.8, 95% CI 4.9–8.6) and DBP by 3.6 mmHg (*β* 3.6, 95% CI 2.3–4.9). Moreover, the prospective cohort study by Lai et al. including 684 pregnant women, and the cross-sectional study by Francis et al. both concluded that vitamin E (in either form) was not significantly associated with BP (13, 25). A recent large sample prospective cohort study showed an inverse J-shaped relationship between dietary vitamin intake and the incidence of new-onset hypertension, with participants in the second to fourth quintiles (Q2-Q4) having the lowest incidence of new-onset hypertension. Compared with Q2-Q4, the incidence of new-onset hypertension increased by 40% in Q1 participants (OR 1.4, 95% CI 1.29–1.52), and by 18% in Q5 (OR 1.18, 95% CI 1.09–1.29) (17). The controversial results in these studies may be explained by differences in study design, sample size, study population, and confounding factors. However, what is more, important is that several different forms of vitamin E are known both *in vivo* and in dietary supplementation, and most of these studies have not been studied separately.

Based on previous studies, the present study is the first to individually investigate the association of GTSC with BP using cross-sectional data from a large sample, adding more evidence to the field. Our study has several important findings. First, our study found that GTSC was linearly and positively correlated with SBP, DBP, and the prevalence of hypertension, with a trend toward higher BP and hypertension prevalence with higher GTC concentrations. It is not clear the specific mechanism by which GTSC increases SBP, DBP, and the prevalence of hypertension, but the following mechanisms might explain it. Firstly, GTC has a dual role in oxidative stress. GTC is known to have antioxidant and anti-inflammatory effects, but some studies indicated that excess GTC could promote the production of nitric oxide, an inflammatory mediator through its oxidation products, leading to an enhancement of cellular immune response and an increase of lipid peroxidation (26). Studies have proven that endothelial damage, vascular dysfunction, cardiovascular remodeling, renal dysfunction, immune cell activation, and systemic inflammation during oxidative stress underlie the pathophysiology of hypertension (27–30). Another possible mechanism is that GTC could increase the excitability of the sympathetic nervous system. This hypothesis was supported by a randomized clinical trial in which heart rate was significantly increased by either ATC or mixed tocopherol treatment, indicating a central effect of tocopherol (24). However, it contradicts another study, which concluded that the cardiac autonomic nervous system improved after treatment with vitamin E (31).

Second, we noted that alcohol consumption could modify the relationship of GTSC with both SBP and DBP. In participants who consumed alcohol, a potentiation of the GTC-driven increase in SBP and DBP was observed, but not in non-drinking participants. Numerous studies have demonstrated that alcohol consumption is positively associated with BP increase, even in small amounts (32–34). The potential mechanisms responsible for this outcome are complex and varied, including effects on the central nervous system, inhibition of the vagal, excitation of the sympathetic nerve, activation of the renin-angiotensin-aldosterone system, increased cortisol secretion, insulin resistance and impaired glucose tolerance, oxidative stress, and impaired endothelial function (35, 36). Overall, our findings encouraged people with high levels of GTSC to lower their GTSC appropriately to control BP, especially those who consume alcohol. However, it is worth noting that the above results are only hypothesis generation and further investigations need to be conducted to support the findings of our study.

Although our study is based on nationally representative U.S. general population data and has a large sample size that minimizes bias and has good extrapolation, we must also acknowledge several important limitations of this study. First, this study was a retrospective cross-sectional design, and thus no causal inferences could be made about the association of GTSC with SBP, DBP, and the prevalence of hypertension, which is an inherent drawback of all cross-sectional designs. Second, although we adjusted for numerous confounding factors, we still cannot exclude the interference of unknown confounders (e.g., daily lifestyle, medication use) on the study results. In this study, only a single blood test was performed to assess the status of GTC, and repeat sampling may be required to overcome the daily variability of individuals, but this process would become very complex and expensive. Fourth, our study was limited to adults in the United States, so generalization to other countries or age groups requires caution.

## 5. Conclusion

In summary, this study investigated the relationship between GTSC and SBP, DBP, and the prevalence of hypertension in the general U.S. population and found that GTSC was linearly and positively associated with SBP, DBP, and the prevalence of hypertension, and found that this correlation of GTSC with SBP and DBP was more significant in those who consumed alcohol.

## Data Availability

The original contributions presented in the study are included in the article, further inquiries can be directed to the corresponding author.
